# The effect of suicide prevention program for community dwelling elderly

**DOI:** 10.1192/j.eurpsy.2023.799

**Published:** 2023-07-19

**Authors:** K. Kim, B.-H. Yoon, H. Gwon, S. Park

**Affiliations:** Naju National Hospital, Naju, Korea, Republic Of

## Abstract

**Introduction:**

The suicide rate in the elderly population is the highest of all ages in Korea. Suicide prevention programs specialized in the elderly are scarce.

**Objectives:**

We evaluated the effect of the suicidal prevention program named “Nae-an-ae” (means to love oneself ), which was specifically designed for the conditions of the community dwelling elderly.

**Methods:**

The subjects were those who agreed to participate in the Nae-an-ae program among those evaluated as suicide high-risk groups according to the 2021 Jeollanam-do Mental Health Survey. The program consisted of five sessions of simple activities that could be practiced in daily life along with knowledge transfer through education on emotion recognition, stress management, sleep and relaxation, pain and exercise, and depression. This program was conducted by social workers or nurses working at each local community mental health and welfare center. We evaluated the Geriatric Depression Scale-Short Form Korean Version (GDS-SF), suicidal ideation, satisfaction with life scale (SWLS) and brief resilience scale (BRS) which were measured before and after the program and compared them with the control group.

**Results:**

A total of 276 participated in the program, 226 were in the control group. In the program participating group, the frequency of suicidal ideation was significantly decreased from 36.2% to 11.6% after the program. GDS-SF, SWLS and BRS were significantly decreased in active group than control group.

**Image:**

**Figure:**
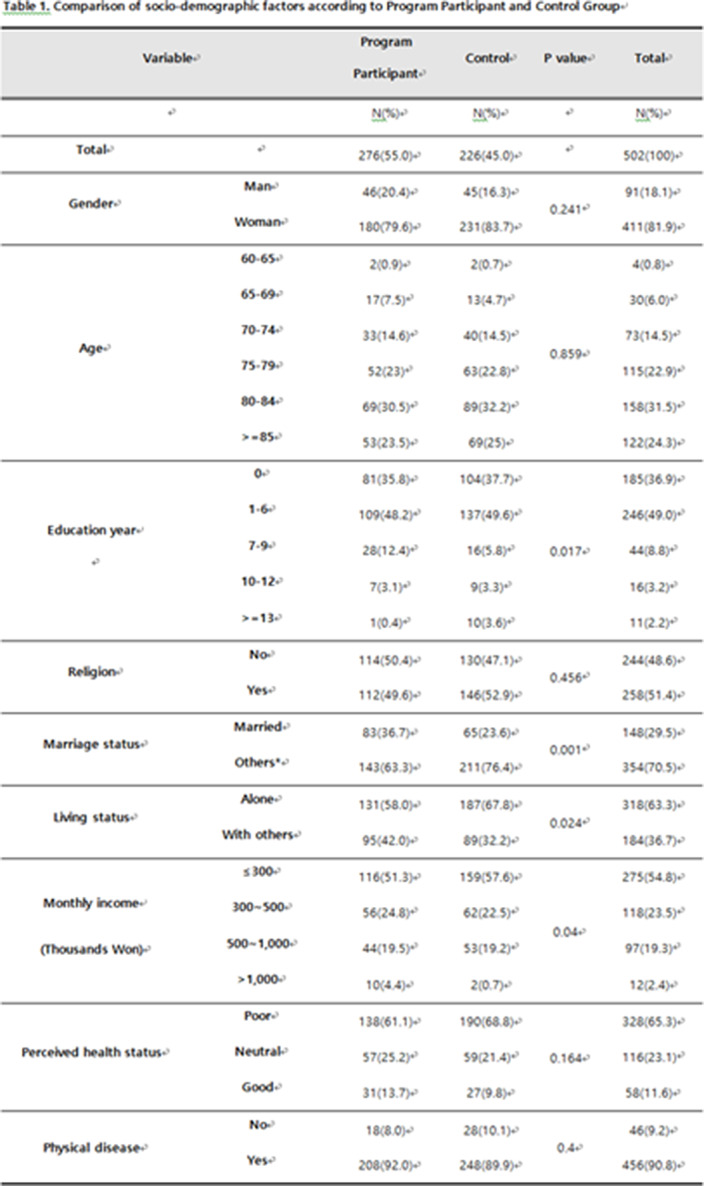

**Image 2:**

**Figure:**
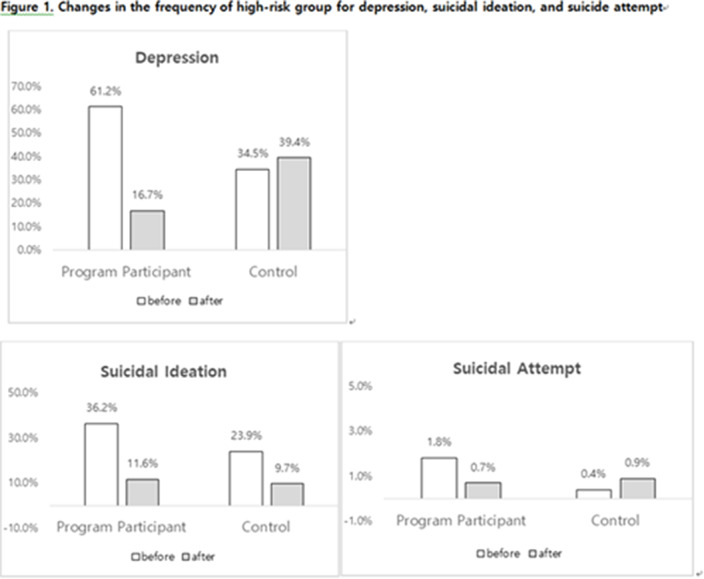

**Image 3:**

**Figure:**
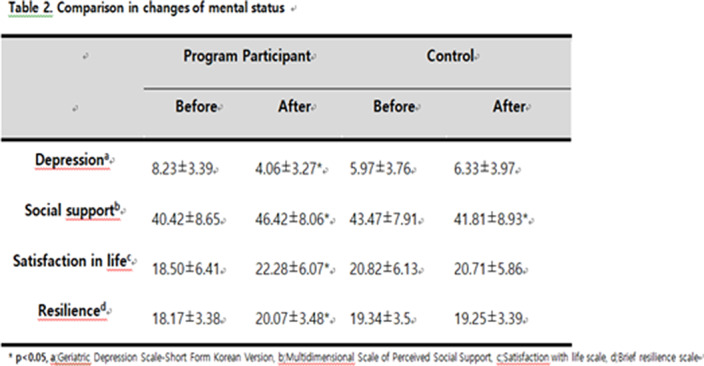

**Conclusions:**

These findings showed that “Nae-an-ae” program was found to affect not only the control of suicide risk factors such as depression but also positive factors such as life satisfaction and resilience.

**Disclosure of Interest:**

None Declared

